# The role of NbTMP1, a surface protein of sporoplasm, in *Nosema bombycis* infection

**DOI:** 10.1186/s13071-021-04595-8

**Published:** 2021-01-25

**Authors:** Shiyi Zheng, Yukang Huang, Hongyun Huang, Bin Yu, Ni Zhou, Junhong Wei, Guoqing Pan, Chunfeng Li, Zeyang Zhou

**Affiliations:** 1 State Key Laboratory of Silkworm Genome Biology, Southwest University, Chongqing, 400715 China; 2 Chongqing Key Laboratory of Microsporidia Infection and Control, Southwest University, Chongqing, 400715 China; 3 College of Life Sciences, Chongqing Normal University, Chongqing, 401331 China; 4 Affiliated Jinhua Hospital, Zhejiang University of Medicine–Jinhua Municipal Central Hospital, Jinhua, 321000 Zhejiang China

**Keywords:** *Nosema bombycis*, Membrane protein, Localization, RNAi, Monoclonal antibody

## Abstract

**Background:**

*Nosema bombycis* is a unicellular eukaryotic pathogen of the silkworm, *Bombyx mori*, and is an economic and occupational hazard in the silkworm industry. Because of its long incubation period and horizontal and vertical transmission, it is subject to quarantine measures in sericulture production. The microsporidian life-cycle includes a dormant extracellular phase and intracellular proliferation phase, with the proliferation period being the most active period. This latter period lacks spore wall protection and may be the most susceptible stage for control.

**Methods:**

In order to find suitable target for the selective breeding of *N. bombycis*-resistant silkworm strains, we screen highly expressed membrane proteins from the transcriptome data of *N. bombycis*. The subcellular localization of the candidate protein was verified by Indirect immunofluorescence analysis (IFA) and immunoelectron microscopy (IEM), and its role in *N. bombycis* proliferation was verified by RNAi.

**Results:**

The *N. bombycis* protein (NBO_76g0014) was identified as a transmembrane protein and named NbTMP1. It is homologous with hypothetical proteins NGRA_1734 from *Nosema granulosis*. NbTMP1 has a transmembrane region of 23 amino acids at the N-terminus. Indirect immunofluorescence analysis (IFA) results suggest that NbTMP1 is secreted on the plasma membrane as the spores develop. Western blot and qRT-PCR analysis showed that *NbTMP1* was expressed in all developmental stages of *N. bombycis* in infected cells and in the silkworm midgut. Downregulation of *NbTMP1* expression resulted in significant inhibition of *N. bombycis* proliferation.

**Conclusions:**

We confirmed that NbTMP1 is a membrane protein of *N. bombycis.* Reduction of the transcription level of *NbTMP1* significantly inhibited *N. bombycis* proliferation, and this protein may be a target for the selective breeding of *N. bombycis*-resistant silkworm strains. 
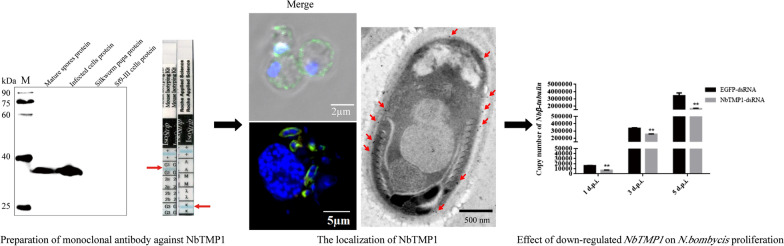

## Introduction

Microsporidia are obligate intracellular parasitic eukaryotes with a wide range of hosts, infecting almost all vertebrates and invertebrates, including humans [1–3]. These spore-forming unicellular parasites have a unique ultrastructure and life-cycle [3]. They were originally considered to be primitive organisms, such as protozoans or protists [4]. However, microsporidia are now considered to be related to fungi or perhaps to be a sister branch of fungi that has lost many genes and undergone genome compression, possibly due to their adaptation to intracellular parasitism [5–8]. The lack of mitochondria is taken as evidence that microsporidia are ancient eukaryotes [9]. As a result of the loss of many metabolic pathways, microsporidia must obtain nutrients from the host and export the cytotoxic compounds through the cell membrane [10–12]. Microsporidia have a distinctive mechanism for infecting host cells. The polar tube is ejected when microsporidia are stimulated by suitable conditions, and the infective sporoplasm is transferred into the host cells through the polar tube prior to proliferation [13]. The sporoplasm can also adhere to host cells and enter the host cells by phagocytosis [14]. The membrane proteins of the sporoplasm then interact with the cytoplasma content of host cell.

*Nosema bombycis*, the first microsporidian to be recognized, by Nageli in 1857, causes silkworm pébrine disease by vertical and horizontal transmission [15]. The prevention and control of *N. bombycis* is an important focus of sericulture research. In this study, we identified a transmembrane protein (NBO_76g0014) of *N. bombycis*, which we designated NbTMP1. We cloned and expressed recombinant protein NbTMP1 and prepared monoclonal antibodies. We also characterized the subcellular localization of NbTMP1 in *N. bombycis* and explored its function in *N. bombycis* proliferation.

## Materials and methods

### Preparation of *N. bombycis* and cell cultivation

Mature spores of *N. bombycis* CQ1, obtained from the China Veterinary Culture Collection Center (CVCC No. 102059), were isolated from infected silkworm pupae and purified by Percoll density gradient centrifugation (21,000 *g*, 40 min) [16]. Cells of ovarian cell line *Spodoptera frugiperda *(Sf9-III) in Sf-900 III™ SFM were purchased from Thermo Fisher Scientific (Waltham, MA, USA) and cultured in Sf-900™ III SFM medium at 28 °C.

### *NbTMP1* sequence analysis and amplification of the open reading frame

NbTMP1 (GenBank Accession No. EOB13409.1), which is a highly expressed protein in the early stages of infection, was screened from the transcriptome data of *Bombyx mori* infected with *N. bombycis*. The amino acid sequence of NbTMP1 was submitted to the SignalP 5.1 server (http://www.cbs.dtu.dk/services/SignalP/) and the TMHMM server v.2.0 (http://www.cbs.dtu.dk/services/TMHMM/) for signal peptide and transmembrane domain predictions. The molecular weight and isoelectric point (pI) were predicted using the ExPASy server (http://web.expasy.org/compute_pi/), and the protein function domain was predicted using SMART (http://smart.embl-heidelberg.de/). BLAST (https://blast.ncbi.nlm.nih.gov/) was used to analyze the multiple sequence alignment. We cloned the extramembranous region of* NbTMP1* using the forward primer 5′-GGATCCATGTTCAAGTCTAGTGATGA-3′ containing a *Bam*HI restriction site (underlined bases) and the reverse primer 5′-GTCGACCTTATCATTTTCATTATTTCCC-3′ containing a *Sal*I restriction site (underlined bases). Complementary DNA (cDNA) was obtained by reverse transcription of RNA from the Sf9-III cells (Thermo Fisher Scientific) infected with *N. bombycis* and used as the PCR template. The PCR products were purified with the Gel Extraction Kit of Omega Bio-tek, Inc. (Norcross, GA, USA) and integrated into the pET-28 vector, following which the vectors were transformed into competent cells of *Escherichia coli* strain DH5α. The positive pET-28-*NbTMP1* recombinant vectors were sequenced by Sangon (Shanghai, China).

### Recombinant protein expression, monoclonal antibody preparation and immunoblotting

The identified pET-28-*NbTMP1* vector was transformed into *E. coli* strain Rosetta for expression. The recombinant bacteria were induced for 4 h at 37 °C with 0.5 mM isopropyl-β-D-thiogalactopyranoside (IPTG) in LB medium. The target protein was purified with nickel-chelating affinity chromatography (Roche Applied Science, Penzberg, Germany). All animal experiments were approved by the Laboratory Animals Ethics Review Committee of Southwest University (Chongqing, China) under Permit Number AERCSWU2017-7. The mice were maintained according to the recommendations of the committee, and food and water were provided ad libitum. Female BALB/c mice (6- to 8 weeks-old) were injected subcutaneously four times with rNbTMP1 (100 μg/mouse) mixed with Freund’s complete/incomplete adjuvant (1:1; Sigma-Aldrich, St. Louis, MO, USA). The injection interval was 7 days. Monoclonal antibodies (mAbs) were produced and screened as previously described [17]. The mAb immunoglobin G (IgG) subtypes were detected using the Mouse Monoclonal Antibody Isotyping Kit (Roche Applied Science) according to manufacturer’s recommendations.

For immunoblotting, the proteins of mature spores and infected cells were prepared by the glass bead breaking method [18], isolated by sodium dodecyl sulfate-polyacrylamide gel elctrophoresis and transferred to PDVF membranes (Roche Applied Science). After blocking in 5% skim milk diluted in TBST (150 mM NaCl, 20 mM Tris-HCl, 0.05% Tween-20), the membrane was incubated for 1 h in anti-NbTMP1 ascites (1:1000). After washing three times, the membrane was incubated with goat anti-mouse IgG (1:5000; Sigma-Aldrich) conjugated with horseradish peroxidase (HRP) label. Finally, the protein bands were visualized with ECL Plus Western Blotting Detection Reagents (Bio-Rad, Hercules, CA, USA).

### Silkworm midgut paraffin section

Newly molted fifth instar silkworm larvae were starved for 1 day and then fed mulberry leaves coated with spores (1 × 10^7^ spores per larva). At 4 days post-infection (dpi), the infected silkworms were killed, and the midguts were collected and stored at − 80 °C. For embedding, the midguts were first washed three times, 15 min each time, in phosphate buffered saline (PBS: 137 mM NaCl, 10 mM Na_2_HPO_4_, 2 mM KH_2_PO_4_ and 2.7 mM KCl), following which the midguts were fixed in 4% paraformaldehyde for 24 h. The sections were prepared as previously described [19]. After deparaffination, the sections were boiled in sodium citrate buffer solution for 10 min and then used in the immunolocalization studies.

### Immunofluorescence analysis

For the immunofluorescence analysis (IFA), the samples were fixed in 4% paraformaldehyde and then washed three times in PBS. The samples were then permeabilized with 0.5% Triton X-100 for 30 min and then blocked with PBS–bovine serum albumin for 1 h at room temperature. Next, the samples were incubated with the primary antibodies, including anti-NbTMP1 ascites (mouse), or with negative serum and Nbβ-tubulin (rabbit) antibody which was used to label meronts of *N. bombycis* [20]. After washing three times with PBS, Alexa 488 conjugate Goat anti-Mouse IgG and Alexa 568 conjugate Goat anti-Rabbit IgG (Thermo Fisher Scientific) were used to detect the bound primary antibodies, and 4′,6-diamidino-2-phenylindole (DAPI) (Thermo Fisher Scientific) was used to label the nucleus. The results were observed under a confocal laser scanning microscope (Olympus, Tokyo, Japan).

### Immunoelectron microscopy study

Infected silkworm midguts were used for the immunoelectron microscopy (IEM) study. Ultrathin sections (70 nm) were prepared as previously described [19] and placed on nickel grids. After blocking with PBS–bovine serum albumin, the grids were incubated with anti-NbTMP1 ascites diluted 1:30 or with negative serum at room temperature for 1 h. The grids were then incubated with gold-conjugated anti-mouse IgG (Aldrich-Sigma) diluted to 1:100. After staining with uranium and lead, the sections were examined and photographed with a JEM-1400 Plus TEM transmission electron microscope (JEOL Co. Ltd., Tokyo, Japan).

### RNA interference of NbTMP1

A 331-bp interferential fragment was selected by BLOCK-iT™ RNAi Designer (http://rnaidesigner.thermofisher.com/rnaiexpress/design.do). The fragment was amplified by the F-RI-NbTMP1-T7 (5′-TAATACGACTCACTATAGGGAGATGTCTCTAGGGCAGATGC-3′) and R-RI-NbTMP1-T7 (5′-TAATACGACTCACTATAGGGAGATCCAGTACGTGTTGCCTG-3′) primers and used as the template for synthesizing double-stranded RNA (dsRNA). The RiboMAX™ Large Scale RNA Production System-T7 (Promega, Madison, WI, USA) was used to obtain the dsRNA. The dsRNA of enhanced green fluorescent protein (EGFP) was used as a negative control and was synthesized as described above [17]. After the Sf9-III cells were cultured in 12-well plates overnight, 2 µg dsRNA of *NbTMP1* or *EGFP* was transfected into the cells. After 4 h, the spores were germinated with 0.1 M KOH and added to the Sf9-III cells (spores:cell, 5:1). Samples of infected cells were collected at 1, 3, and 5 dpi and immediately stored in PBS or TRIzol (Invitrogen, Carlsbad, CA, USA) at − 80 °C.

### Real-time quantitative PCR analysis

A DNA Extraction Kit (Omega Bio-tek, Inc.) was used to extract the genomic DNA (gDNA) of the infected cells. Total RNA Kit II (Omega Bio-tek, Inc.) and EvoScript Universal cDNA Master (Roche Applied Science) were used to prepare the cDNA. Real-time quantitative PCR (qPCR) was amplified by the F-q-Nb TMP1 (5′-CCTATCTCTAAAGACGGT-3′) and R-q-Nb TMP1 (5′-CTTTTTCTATTTTGGCAGCA-3′) primers, and reference gene primers F-q-SSU (5′-CTGGGGATAGTATGATCGCAAGA-3′) and R-q-SSU (5’-CACAGCATCCATTGGAAACG-3′). The transcription levels were calculated by the 2^-△△t^ values method with three replicates. GraphPad Prism v6.01 (GraphPad Software, San Diego, CA, USA) was used to conduct the multiple* t* tests.

The *Nbβ-tubulin* copy number was used to count *N. bombycis*. The gDNA of infected cells samples was analyzed by qPCR. The reaction systems were conducted using the primers: Nbβ-tubulin-qF (5′-AGAACCAGGAACAATGGACG-3′) and Nbβ-tubulin-qR (5′-AGCCCAATTATTACCAGCACC-3′). The standard template used was described in previous research [17]. The standard curve covered six orders of magnitude (1.3 × 10^2^–10^7^).

## Results

### Sequence characteristics and immunoblot analysis of NbTMP1

**Fig 1.**
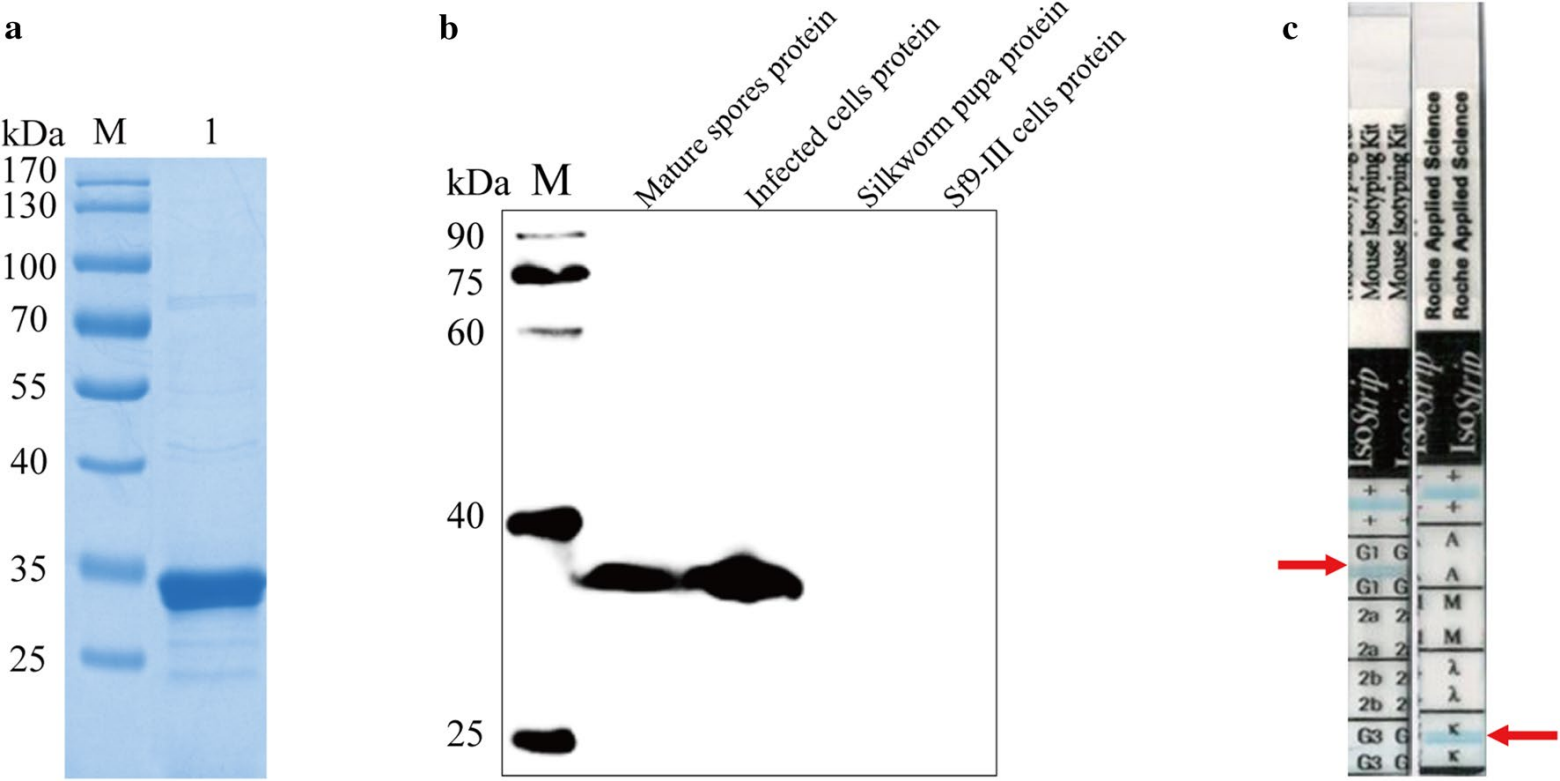
*Nosema bombycis* protein (NBO_76g0014) was identified as the transmembrane protein NbTMP1. **a** Purification of recombinant NbTMP1. Lanes:* M* PageRuler Prestained protein ladder marker,* 1* recombinant NbTMP1. **b** Specificity of the F12 monoclonal antibody. Proteins extracted from mature spores, infected Sf9-III cells, healthy silkworm pupa and Sf9-III cells were detected by western blotting using F12 ascites against NbTMP1. * M* Protein marker (Transgene, Shanghai, China). **c** Subtype identification of F12. The red arrow on the left indicates that the heavy-chain subtype was immunoglobulin G subtype 1 (IgG1), and the right arrow points to the light-chain subtype, kappa (*κ*). The blue line of the ‘+’ region is the control lane

Due to the loss of many metabolic pathways, microsporidia must obtain nutrients and energy from the host through the cell membrane. In order to facilitate the control of *N. bombycis* in the cell, we attempted to screen highly expressed membrane proteins from the transcriptome data of *N. bombycis*. NbTMP1, a hypothetical membrane protein, was highly expressed in the transcriptome data of *Bombyx mori* infected with *N. bombycis*, suggesting that it may be involved in the proliferation of *N. bombycis*. Sequence analysis showed that NbTMP1 is 43% homologous to the hypothetical protein NGRA_1734 (GenBank Accession No. KAF9762805.1) in *Nosema granulosis* [21]. *NbTMP1* contained a complete open reading frame (ORF) that was 732 bp in length and encoded a polypeptide of 243 amino acids with a transmembrane domain. The protein has a calculated molecular weight of 27.59 kDa and a theoretical pI value of 9.92. It does not have a signal peptide and typical functional domains. The recombinant NbTMP1 protein was expressed as a soluble protein of about 30 kDa (Fig. 1a) and was purified to prepare the monoclonal antibody F12.

Western blot analysis with F12 ascites revealed a unique positive band in mature spores and in the proteins of infected cells (Fig. 1b). The band is about 35 kDa, which is more than the molecular weight of NbTMP1, possibly due to post-translational modification, such as phosphorylation and glycosylation, of the native NbTMP1. Bioinformatics analysis showed that there are two glycosylation sites and 20 phosphorylation sites. Since the recombinant protein lacks a transmembrane domain, the molecular mass of the recombinant NbTMP1 and the native protein differed. The Mouse Monoclonal Antibody Isotyping Kit (Roche Applied Science) was used to identify the subtypes of monoclonal antibody F12, and the test strip demonstrated that the subtypes of F12 were IgG1-κ (Fig. 1c).

### Subcellular localization of NbTMP1

**Fig 2.**
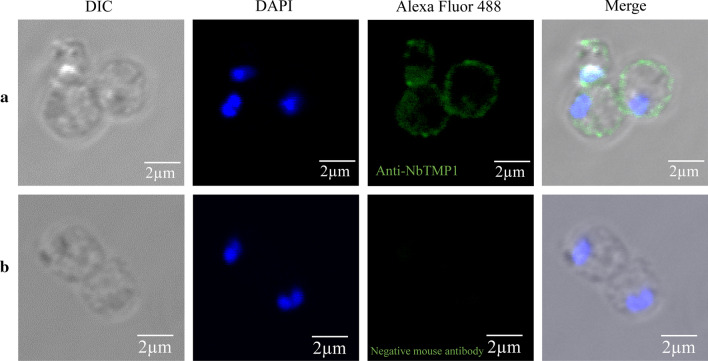
Localization of NbTMP1 in sporoplasm. The sporoplasm obtained after germination subject to immunofluorescence analysis. The sporoplasm was labeled with anti-NbTMP1 (**a**) or negative mouse antiserum (**b**). The NbTMP1 signal is shown as green staining (Alexa Fluor 488), and nuclei are shown with blue staining (DAPI).* DAPI* 4′,6-Diamidino-2-phenylindole, * DIC* Differential interference contrast

**Fig 3.**
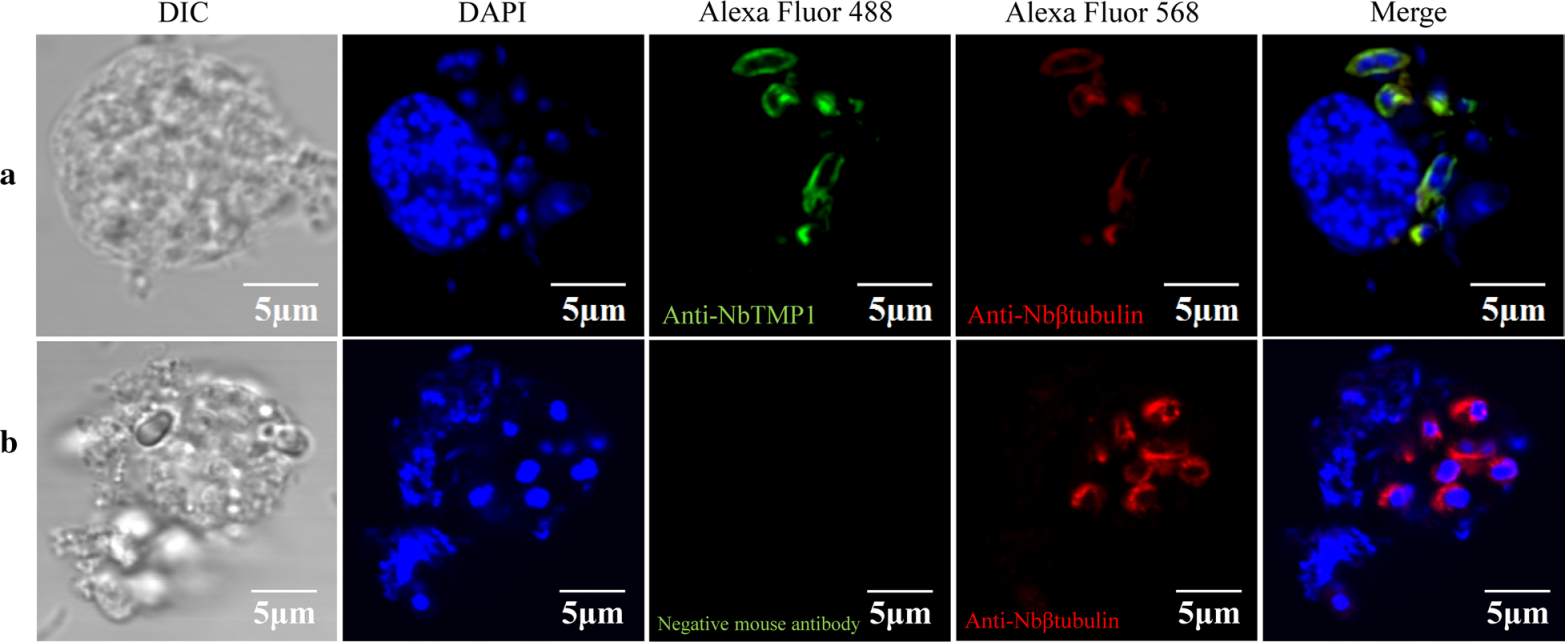
Subcellular localization of NbTMP1 in infected Sf9-III cells. The location of NbTMP1 was imaged with confocal microscopy images in meronts. **a** Infected cells were treated with F12 (mouse antiserum, anti-NbTMP1) and Nbβ-tubulin antiserum (rabbit antiserum). **b** Infected cells were treated with negative mouse antiserum and Nbβ-tubulin antiserum (rabbit antiserum). Alexa Fluor 488 (green) or Alexa Fluor 568 (red) were incubated with mouse antiserum and rabbit antiserum, respectively. The nuclei were labeled with DAPI (blue)

**Fig 4.**
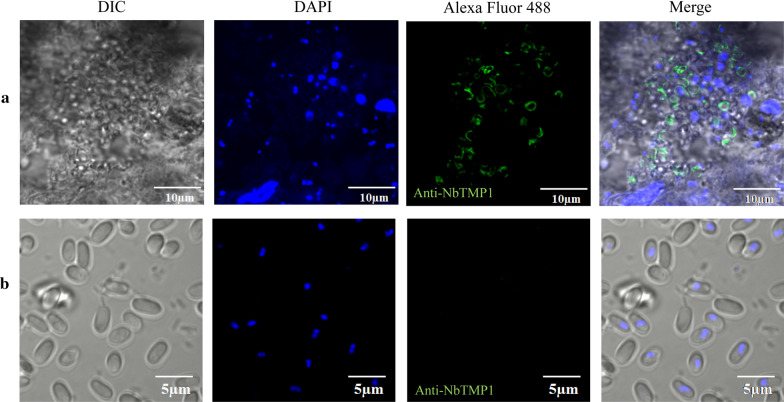
Subcellular localization of NbTMP1 in infected silkworm midguts (**a**) and mature spores (**b**). The midgut tissue section and unsliced mature spores were labeled with DAPI (nuclei) and anti-NbTMP1 (green)

**Fig 5.**
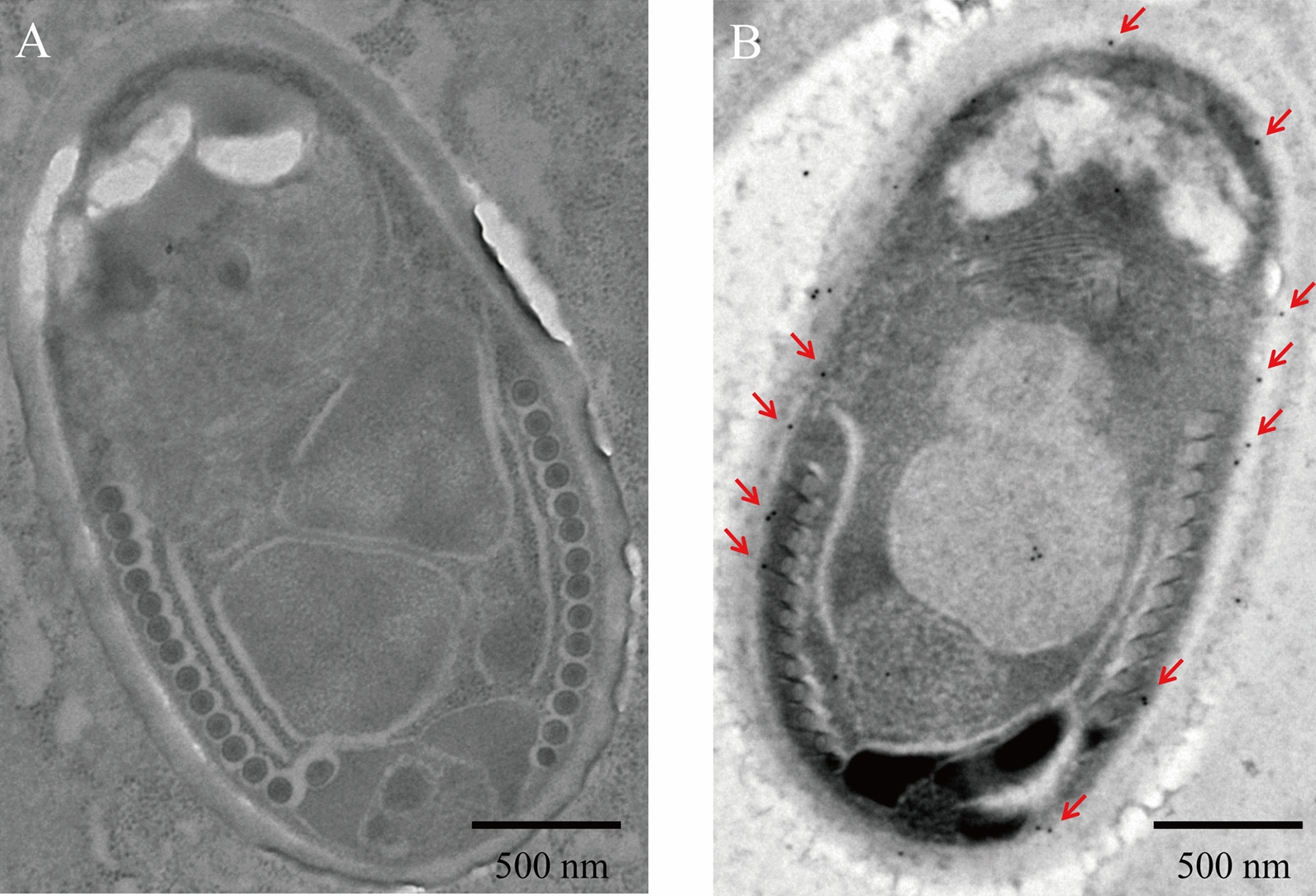
Immunoelectron microscopy of NbTMP1 in spores of infected midgut. **a** Negative control, **b** spore showing the presence of gold labeled anti-NbTMP1 on the plasma membrane. The red arrow indicates the gold label. Scale bars 500 nm

To study the subcellular localization of NbTMP1, we performed IFA with the F12 ascites. Sporoplasm was obtained by allowing mature spores to germinate in 0.1 M KOH. The IFA indicated that NbTMP1 is localized on the plasma membrane of the sporoplasm (Fig. 2). We used the Nbβ-tubulin antibody to label meronts and noted that NbTMP1 co-located with Nbβ-tubulin in the proliferation stage (Fig. 3). The subcellular localization of NbTMP1 was verified by examing paraffin sections of infected tissues. The fluorescence signal of NbTMP1 was consistent with the contour of mature spores (Fig. 4a), but there was no fluorescence signal in mature spores that had not been sectioned (Fig. 4b). As antibodies cannot penetrate the spore wall of *N. bombycis*, this observation also implies that NbTMP1 is located on the plasma membrane. IEM analysis further demonstrated that NbTMP1 is located on the membrane of *N. bombycis* (Fig. 5). This result indicates that NbTMP1 may be secreted to the plasma membrane during the development of *N. bombycis*.

### Transcriptional profile of *NbTMP1* in infected cells and midguts

**Fig 6.**
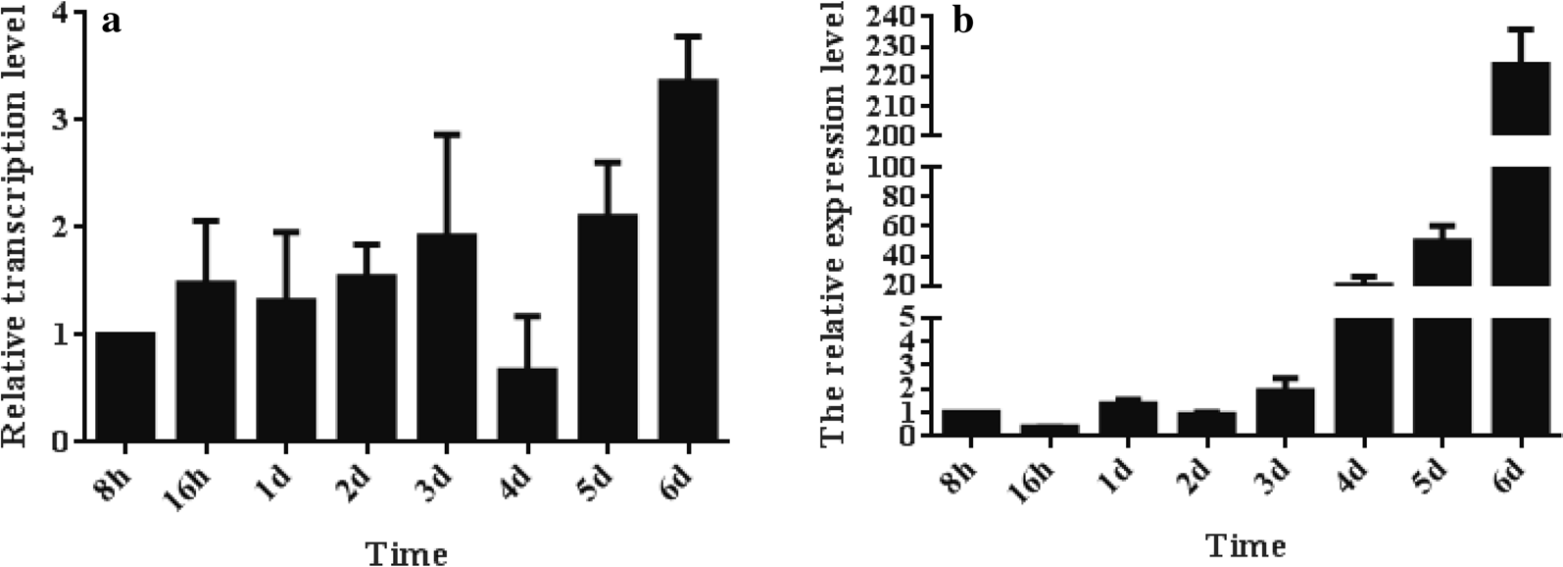
Transcription analysis of *NbTMP1*. *N. bombycis* small subunit ribosomal ribonucleic acid was used as an internal control. The relative expression level of *NbTMP1* is presented relative to expression at 8 h post-infection. **a** Transcription level of *NbTMP1* in infected Sf9-III cells, **b** transcription level of *NbTMP1* in infected silkworm midguts. Vertical bars show the mean ± standard error (*n *= 3)

After *N. bombycis* infected the Sf9-III cells, the expression of *NbTMP1* increased from the 1 dpi to 6 dpi. The drop in *NbTMP1* expression on 4 dpi may be due to the formation of mature spores (Fig. 6a). In the midguts of infected silkworms, *NbTMP1* showed low expression level in the pre-infection phase, with expression increasing continuously after 4 dpi (Fig. 6b). On 4 dpi, *N. bombycis* began a new round of host infection, and the transcript profile of *NbTMP1* suggested that it may play a role in spore infection.

### RNA interference of *NbTMP1* inhibited* N. bombycis*’ proliferation

**Fig 7.**
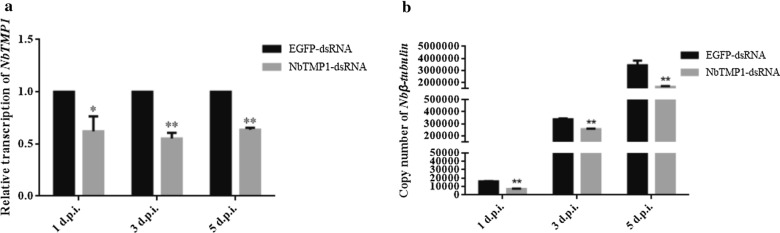
Effect of downregulated *NbTMP1* on *N. bombycis* proliferation via RNA interference. **a** The transcription levels of *NbTMP1* in infected Sf9-III cells with NbTMP1-dsRNA or EGFP-dsRNA at 1, 3 and 5 days post infection (*d.p.i.*). **b** The number of *N. bombycis* in Sf9-III cells. The copy number of *Nbβ-tubulin* indicates the number of *N. bombycis*. Asterisks represent statistically significant differences at *P < 0.05 and **P < 0.01). Scale bars represent the standard deviation of three independent repeats. * EGFP* Enhanced green fluorescent protein

The effects of RNA interference (RNAi) were analyzed using qPCR. Following the addition of the dsRNA, the transcription level of *NbTMP1* was significantly downregulated in the experimental groups (NbTMP1-dsRNA) (Fig. 7a). Since *Nbβ-tubulin* is a housekeeping and conserved gene of *N. bombycis*, its copy number was used to reflect the number of *N. bombycis* in the two groups [17]. The qPCR results showed that *N. bombycis* began to proliferate from 1 to 5 dpi in the mock groups (EGFP-dsRNA). However, the pathogen load was remarkably lower in the experimental groups (Fig. 7b). These results suggest that NbTMP1 plays an important role in the proliferation of *N. bombycis*.

## Discussion

Membrane protein is rarely studied in microsporidia. In *Trachipleistophora hominis*, plasma membrane-located purine nucleotide transport proteins (NTTs) are key components of the process involved in stealing ATP from hosts [11]. EhSSP1 was identified as *Encephalitozoon hellem* sporoplasm surface protein 1; this protein binds to human foreskin fibroblasts and is associated with the final step of invasion on the invasion synapse [22]. NbTMP1 was predicted to have a transmembrane domain, suggesting that it may be located on the membrane of the spore. The IFA and IEM results proved that NbTMP1 actually did localize on the membrane of sporoplasm and spores. The expression profiles* in vitro* and* in vivo* and the western blot results showed that NbTMP1 is present in all stages of the *N. bombycis* life-cycle. Although we showed that downregulation of NbTMP1 expression can inhibit the proliferation of *N. bombycis*, the specific mechanism remains unknown.

There are two main hypotheses on how microsporidia infect host cells. One is that the microsporidia eject a polar tube, penetrate the membrane of new host cells and then deliver the contents into the cytoplasm of the host cell; the other is that the microsporidia gain access to the host cell by phagocytosis, and then the polar tube is used to escape the phagosome and to infect the host cytoplasm [13]. Sporoplasm is the earliest stage of microsporidia infection of host cells. The mechanism used by microsporidia to transmit genetic information after infecting a host cell is unclear. However, surface proteins, such as NbAQP, NbSWP9 and NbSWP7, can play important roles in the process of spore germination and infection [23, 24]. NbTMP1 is located on the plasma membrane of the sporoplasm, suggesting that it may be involved in the proliferation of spores in cells.

Understanding the protein functions of *N. bombycis* has been hampered by the lack of stable and reliable gene manipulation methods. RNAi has been used in fungi, including *Heterosporis saurida* and *Nosema ceranae*, to study protein functions [25, 26]. *Nosema bombycis* possesses all of the genes required for RNA silencing and* in vivo* function [27]. A stable RNAi strategy was previously established utilizing an* in vitro* transcription system and lipofection in Sf9-III infection [28]. RNAi has been successfully used to develop* Bombyx mori* nucleopolyhedrovirus (anti-BmNPV)-resistant silkworms [29]. RNAi can also be used as a strategy for breeding *N. bombycis*-resistant silkworms, and NbTMP1 is a potential target of RNAi. Single-chain antibodies are also a strategy for breeding *N. bombycis*-resistant silkworms. In *Anopheles*, transposon-mediated transformation was used to generate m1C3, m4B7 and m2A10 single-chain antibodies (scFvs), and the transgenic mosquitoes expressing the scFv gene had significantly lower infection levels of *Plasmodium falciparum* [30, 31]. The transgenic Sf9-III cells, which express single-chain antibodies of SWP12 of *N. bombycis*, effectively inhibit the proliferation of *N. bombycis* in cells [17]. In this study, we obtained the monoclonal antibody (F12) of NbTMP1, and in future investigations we plan to evaluate the performance of this single-chain antibody for breeding of *N. bombycis*-resistant silkworms. In follow-up studies, we will try to express dsRNA of *NbTMP1* and scFv-F12 in silkworm individuals by transgenic technology to verify the function of NbTMP1 and evaluate the resistance of transgenic silkworms to *N. bombycis*.

## Conclusions

We identified a novel membrane protein in the microsporidium *N. bombycis*. NbTMP1 was localized on the plasma membrane of the sporoplasm, meronts and spores, and its proliferation was significantly inhibited by RNAi. This membrane localized protein is important for pathogen development, and may be a potential target for construction of *N. bombycis*-resistant silkworms.

## Data Availability

Data supporting the conclusions of this article are included within the article. All data are fully available without restriction upon reasonable request.

## Abbreviations

IFA: Indirect immunofluorescence analysis
IEM: Immunoelectron microscopy
Sf9-III cells: Ovarian cell line of *Spodoptera frugiperda*
RNAi: RNA interference
qPCR: Real-time quantitative PCR
